# GeneXpert or chest-X-ray or tuberculin skin testing for household contact assessment (GXT): protocol for a cluster-randomized trial

**DOI:** 10.1186/s13063-022-06587-0

**Published:** 2022-08-02

**Authors:** Anete Trajman, Menonli Adjobimey, Mayara Lisboa Bastos, Chantal Valiquette, Olivia Oxlade, Federica Fregonese, Dissou Affolabi, Marcelo Cordeiro-Santos, Renato T. Stein, Andrea Benedetti, Dick Menzies

**Affiliations:** 1 McGill University, Montreal, Canada; 2 Universidade Federal Do Rio de Janeiro, Rio de Janeiro, Brazil; 3 Centre National Hospitalier Universitaire de Pneumo-Phtisiologie, Cotonou, Benin; 4 Research Institute of the McGill University Health Center, Montreal, Canada; 5 Fundação de Medicina Tropical Dr. Heitor Vieira Dourado, Manaus, Brazil; 6 Universidade Do Estado Do Amazonas, Manaus, Brazil; 7 Programa PROADI-SUS, Hospital Moinho de Vento, Porto Alegre, Brazil; 8 Escola de Medicina, Pontifícia Universidade Católica RGS, Porto Alegre, Brazil

**Keywords:** Latent tuberculosis, GeneXpert, Chest X-ray, Tuberculin skin testing, Household contacts, Cascade of care

## Abstract

**Background:**

The World Health Organization recommends tuberculosis (TB) preventive treatment (TPT) for all people living with HIV (PLH) and household contacts (HHC) of index TB patients. Tests for TB infection (TBI) or to rule out TB disease (TBD) are preferred, but if not available, this should not be a barrier if access to these tests is limited for high-risk people, such as PLH and HHC under 5 years old. There is equipoise on the need for these tests in different risk populations, especially HHC aged over 5.

**Methods:**

This superiority cluster-randomized multicenter trial with three arms of equal size compares, in Benin and Brazil, three strategies for HHC investigation aged 0–50: (i) tuberculin skin testing (TST) or interferon gamma release assay (IGRA) for TBI and if positive, chest X-Ray (CXR) to rule out TBD in persons with positive TST or IGRA; (ii) same as (i) but GeneXpert (GX) replaces CXR; and (iii) no TBI testing. CXR for all; if CXR is normal, TPT is recommended. All strategies start with symptom screening. Clusters are defined as HHC members of the same index patients with newly diagnosed pulmonary TBD. The main outcome is the proportion of HHC that are TPT eligible who start TPT within 3 months of the index TB patient starting TBD treatment. Societal costs, incidence of severe adverse events, and prevalence of TBD are among secondary outcomes. Stratified analyses by age (under versus over 5) and by index patient microbiological status will be conducted.

All participants provide signed informed consent. The study was approved by the Research Ethic Board of the Research Institute of the McGill University Health Centre, the Brazilian National Ethical Board CONEP, and the “Comité Local d’Éthique Pour la Recherche Biomédicale (CLERB) de l’Université de Parakou,” Benin. Findings will be submitted for publication in major medical journals and presented in conferences, to WHO and National and municipal TB programs of the involved countries.

**Discussion:**

This randomized trial is meant to provide high-quality evidence to inform WHO recommendations on investigation of household contacts, as currently these are based on very low-quality evidence.

**Trial registration:**

ClinicalTrials.gov NCT04528823.

## Strengths and limitations of this study

- This is a pragmatic multicenter trial that will be conducted in high burden low- and middle-income countries in different types of healthcare facilities. This increases the generalizability of findings.
- This randomized trial is meant to provide high-quality evidence to inform WHO recommendations on investigation of household contacts; currently, these are based on very low-quality evidence.
- The routine use of the GeneXpert molecular test for TB (GX) as a replacement for chest X-ray in the current diagnostic algorithms is novel. This will provide information on a potential solution to a major roadblock in current management of household contacts.
- Different outcomes will be assessed, all of which are relevant for stakeholders. These include the cost-effectiveness of each strategy, the proportion of contacts with confirmed tuberculosis, and the proportion of contacts initiating tuberculosis preventive treatment. The completion of TPT and occurrence of treatment associated adverse events with each strategy will be secondary outcomes.
- A potential limitation is that 40% of adults and an even higher proportion of children may be unable to provide sputum in strategy 2, resulting in many patients assigned to strategy 2 being investigated as if they had been randomized to strategy 1. However, the proportion unable to provide sputum samples is an important finding that will be informative for policy makers. The yield of GX in those who produce a sputum sample will be assessed in secondary analyses.

## Introduction

Tuberculosis (TB) was responsible for 1.3 million deaths in HIV-negative persons in 2020, [1] despite being preventable and curable. Prevention of the disease through treatment of TB infection (TBI) is a key strategy for TB elimination, [2] as nearly 1/4 of the global population has TBI [3] of whom approximately 10% will develop the disease, maintaining the transmission chain. For these reasons, the United Nations’ High-Level Meeting (UN-HLM) held in 2018 recommended to provide preventive TB treatment (TPT) to 30 million people by 2022. This included 6 million persons living with HIV (PLHIV), 4 million children under 5 years of age who were contacts of pulmonary TB patients and 20 million other contacts [4]. The goal for PLHIV was attained and some progress has been made to reach the goal among children, but up to December 2020, only 0.3 million contacts over 5 years of age completed TPT. This represents only 1.6% of the UN-HLM target [1].

To increase uptake of TPT worldwide, shorter and safer rifamycin-based regimens have been adopted to replace the current standard of 6–9 months of isoniazid [5]. However, treatment adherence represents only the tip of the iceberg of the cascade-of-care for TBI. Many losses occur along all the other steps of the cascade-of-care [6]. These include identifying those eligible for investigation, placing and reading a tuberculin skin test (TST), or performing an interferon-gamma release assay (IGRA), ruling out TB disease through medical evaluation and a chest radiograph (CXR) in those with a positive TST or IGRA, and finally the prescription of TPT [6]. A systematic review reported that less than 30% of those eligible for TPT receive a prescription [6].

Recognizing the real and perceived barriers in large scale implementation of testing for TBI and ruling out TB disease (TBD), the World Health Organization (WHO) recommends that if TST/IGRA or CXR are not available, this should not be a barrier for TPT prescription for those in need [5]. However, performance of TST/IGRA and CXR is recommended where feasible [5]. This is because TST and IGRA identify people at greater risk of progressing to TBD [7]. Since TPT may cause serious adverse events, including fatal hepatotoxicity [8], this should be offered only to those who will benefit. Importantly, systematic reviews and large-scale clinical trials have consistently shown that TST identifies those who will benefit from TPT, even among immunocompromised patients, such as those living with HIV [9–12]. Surveys have found that half of household contacts in low- and middle-income countries [13] and over 2/3 of PLHIV are TST-negative [14], suggesting that the majority of persons who would be treated if TBI testing was not accessible would be exposed to potential harms, without significant benefit [6, 14].

With regard to ruling out TBD, systematic reviews [15, 16] have estimated that sensitivity of symptom screening is close to 80% in PLHIV who are not receiving antiretroviral treatment, but the sensitivity of symptom screening is less than 50% in PLHIV receiving antiretroviral treatment [17]. Although systematic reviews of randomized trials have concluded that TPT does not increase the risk of resistance to rifampicin [18] or isoniazid [19], in all trials included in these reviews, participants underwent CXR to exclude active TBD before initiating TPT.

In summary, current evidence favors the need for a TST (or IGRA) to identify those who will benefit most from TPT and a CXR to exclude disease before offering TPT, but equipoise exists.

## Objectives

To evaluate the effectiveness and cost-effectiveness for TPT initiation of three strategies to investigate household contacts (HHC) of TB index patients in Benin and Brazil:

- 1. Standard care (control arm): will follow current WHO current recommendations for household contacts over 5 years of age, meaning a TST for all, with a CXR for those with a positive TST.
- 2. GeneXpert: follow current WHO current recommendations except CXR is replaced by sputum GeneXpert MTB/RIF® (GX).
- 3. A CXR is done for all household contacts over 5 years of age; TST is not done.

## Methods and analysis

### Study design

This is a superiority, open label cluster-randomized multicenter trial with three arms of equal size; clusters are defined as all HHC of patients with newly diagnosed active pulmonary TB. The first eligible member of the household who provides signed informed consent to participate is randomized to one of the three strategies. All subsequently enrolled members of the household are assigned to the same arm. Randomization is computer-generated and stratified by country, in blocks of variable length.

### Study sites

The coordinating center is based in the McGill TB Centre of the Research Institute of the McGill University Health Centre, Montreal, Canada. Clinics reporting more than 80 TB patients per year were selected in Porto-Novo and Cotonou, Benin and in Rio de Janeiro, Porto Alegre and Manaus, Brazil. Clinics were purposely selected to representative of the diagnostic facilities and capacities available in these countries. Some clinics have all diagnostic facilities on site; others have made administrative arrangements with nearby health facilities for the rapid performance of the needed tests, for study participants.

### Eligibility and inclusion and exclusion criteria

Index patients aged 13 or more in Benin or 14 or more in Brazil with pulmonary TB diagnosed within the past 30 days are approached for permission to invite their HHC to participate. Index cases are classified as microbiologically confirmed or clinically diagnosed. Microbiological confirmation is defined as a positive sputum smear microscopy, GX, or culture for *M. tuberculosis*. Index cases are excluded if they have no eligible HHC or they have confirmed MDR (patients with mono-resistance to INH or rifampin can be included as long as they have documented sensitivity to the other drug).

Eligible HHC are those aged up to 50 years (under 5 years of age are excluded in Benin), not known to have HIV infection, and who have not had a CXR or any TBI test in the past 3 months. For HHC with unknown HIV status, HIV testing is not mandatory but is offered according to National Guidelines [20, 21]. The reason for the upper age limit is the higher risk for severe adverse events (hepatotoxicity) of isoniazid in persons over the age of 50. In Benin, all HHC under 5 are prescribed TPT, regardless of testing; thus, they are not included. In Brazil, current policy for investigation of HHC of all ages is TST, CXR for those with a positive TST; thus, all age HHC up to 50 years are eligible in Brazil. In Benin, pregnant women can be included after the first trimester and undergo CXR—when indicated—with appropriate shielding. In Brazil, female HHC undergo a urine pregnancy test before randomization, and if positive, they are excluded.

### Interventions

All strategies start with symptom screening for cough, fever, night sweats, and weight loss. In all three strategies, the investigations mandated by the protocol are the minimum required. The treating clinical team can, at any time, order additional investigations, such as HIV testing, and prescribe non-TB related treatment, if they feel these are clinically warranted. All non-protocol mandated investigations and treatment are recorded, and the costs added in the calculation of total costs for each strategy, regardless of whether these added costs are borne by the public health system or the patient.

#### ***Strategy 1 (standard investigation or control arm, Fig. ******1******)***

**Fig. 1:**
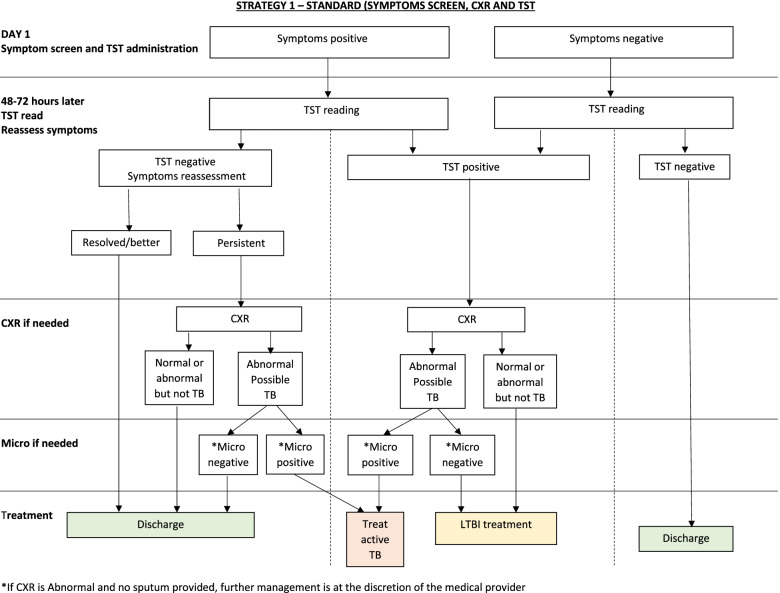
Algorithm of strategy 1 (standard or control arm, based on 2018 WHO algorithm). CXR, chest X-ray; TST, tuberculin skin testing; TB, tuberculosis; LTBI, latent TB infection; Micro, microbiological tests according to NTP guidelines

This strategy is based on the 2018 WHO recommended algorithm for HHC that are HIV-negative and aged ≥ 5 years [5]. Simultaneously with symptom screen, a TST is performed at the first interview. An interferon-gamma release assay (IGRA) would be an acceptable alternative, but in the two countries where this study is conducted, IGRA testing is not accessible in the public health system. Training for TST administration and reading is done during initial site training, if needed. The “mTST tool” [22] is used as a quality assurance tool for TST administration and reading (see link for video on mTST instructions https://www.youtube.com/watch?v = PsBTYiEAKcc&t = 4 s).

The TST induration is read 48 to 72 h after tuberculin injection. The cut-point for a positive TST follows the NTP guidelines in each country. The Brazilian guidelines recommend a second TST 8 weeks later if the first TST is negative. This is not mandated by the study protocol—to be consistent in both countries and with WHO guidelines. However, if providers wish to do this second TST, this can be done, and therapy given if the second TST is at least 10 mm greater than the first TST (defined as conversion). At the time of TST reading (i.e., after 48–72 h), patients with symptoms at the time of initial screening are reassessed for persistence of symptoms.

Participants with negative TST and with no symptoms or resolved symptoms are discharged. If TST is positive or if symptoms persist, a CXR is performed. If the CXR is normal and they have no symptoms, they are recommended to start TPT. If the CXR is abnormal, or normal but TB symptoms persist, they undergo microbiological testing. If the microbiological tests are negative for TBD, then participants are discharged if TST is negative or recommended to start TPT if TST is positive.

#### ***Strategy 2 (GX and TST, Fig. ******2******)***

**Fig. 2:**
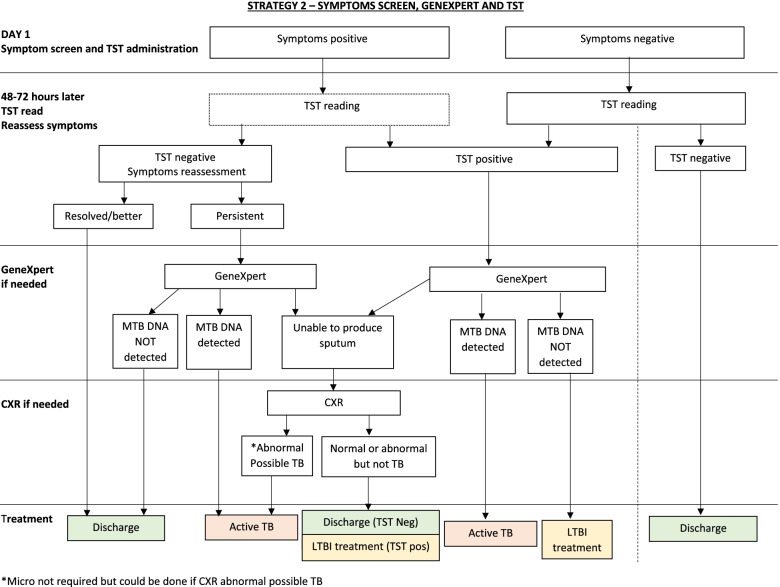
Algorithm of strategy 2 (GeneXpert replaces CXR). CXR, chest X-ray; TST, tuberculin skin testing; TB, tuberculosis; LTBI, latent TB infection; Micro, microbiological tests according to NTP guidelines; MTB, *Mycobacterium tuberculosis*

The key difference from strategy 1 is that GX replaces CXR. Initial steps of symptoms screening and TST administration with symptom re-assessment at time of TST reading 48–72 h later are the same. However, those who are TST positive, or TST negative but with persistent symptoms, have a GX test. It is predicted that even after careful training, approximately 40% of adolescents and adults, and an even higher proportion of children between 5 and 10 years of age, will not be able to produce a sputum sample. In these participants, this could result in missed TBD, so if participants cannot produce a sputum sample, they will have a CXR. If the CXR is abnormal and judged possibly or probably TBD such that microbiologic investigations are necessary, procedures outlined for the other two arms are followed. In a planned secondary analysis, we will examine the proportion of participants in the GX arm (strategy 2) who are unable to produce sputum and (i) are judged to have possible or probable TBD on CXR or (ii) are treated for TBD or (iii) have positive sputum cultures. For children under 5 years of age, sputum induction is performed using the method described by Zar et al. [23].

#### ***Strategy 3 (CXR for all, no TST, Fig. ******3******)***

**Fig. 3:**
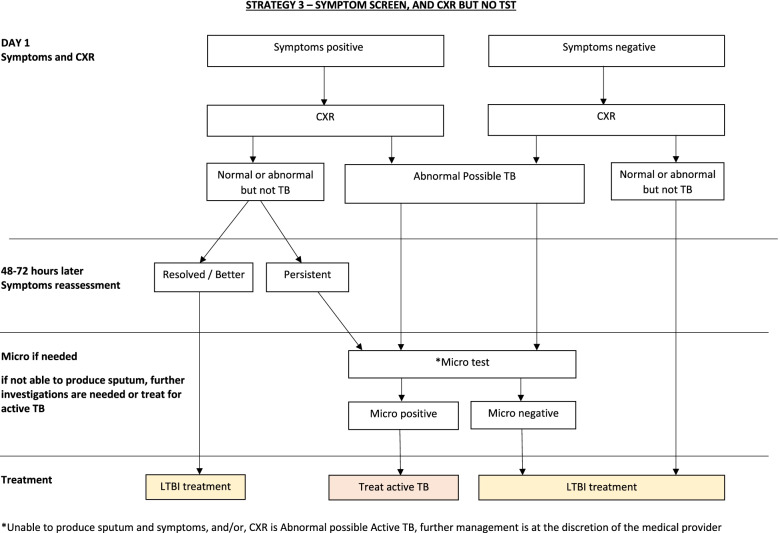
Algorithm of strategy (CXR to all, no TST). CXR, chest X-ray; TB, tuberculosis; LTBI, latent TB infection; Micro, microbiological tests according to NTP guidelines

This group undergoes CXR regardless of the presence of symptoms. Participants with symptoms are re-assessed 2–3 days after the first consultation. If CXR is normal and they do not have persistent symptoms, they are offered TPT. If the CXR is abnormal, or symptoms are persistent, microbiological investigations are done. These investigations follow NTP guidelines, as above. If microbiological investigations are negative, then medical evaluation is done to exclude other respiratory illnesses. If no sputum is obtained for microbiologic investigations, then this is considered a negative microbiologic result. Final decisions on management are at the discretion of the treating team/provider. Once TBD is considered excluded by the treating team, then TPT will be recommended to the HHC.

### Treatment

In Benin, 3 months of rifampicin and isoniazid (3HR) in fixed-dose combination is being used, as this is the continuation phase treatment of TBD and thus available in all clinics. In Brazil, 6 months of isoniazid (6H) was programmatically the treatment of choice at the beginning of the study, since February 2022, 12 weekly doses of rifapentine and isoniazid (3HP) is the first choice. Four months of rifampicin (4R) is recommended for those over 50 years of age, under 10 years of age and for those with suspected or confirmed chronic liver disease. To decrease the risk of treatment especially in strategy 3, we offer 4R (or 3HP since February 2022) for all participants in whom TPT is indicated in all strategies.

### Data gathering

#### Reporting and follow-up

Case report forms (CRF) are completed for identification of index patients and each participating HHC. For HHC, CRF for randomization, study procedures and follow-up are also completed. The study personnel ensure that the protocol-mandated procedures are followed for each strategy, but any medical decision by the treating team is allowed, although recorded. Because this was intended to be a pragmatic trial, we adopted procedures that were in place in the sites and used routinely to enhance adherence. Thus, if the participant misses one procedure or appointment, the study team tries to make contact once, by telephone. When the investigation of the HHC is completed, this is reported using a specific “investigation completed” CRF. If the protocol-mandated investigation is not completed within 3 months after randomization, this is considered a failure of the strategy, and the same CRF is completed. Participants who receive TPT are followed by the clinical staff as per national guidelines. If started on TPT, then outcomes of TPT are recorded on a specific CRF completed at the end of therapy. If an AE occurs during TPT, that is recorded separately on a different AE specific CRF. If at any time during study follow-up TB disease is diagnosed, or death occurs, these are recoded on TBD or death CRFs respectively.

Treatment adherence is based on participants’ report and medical records. Death from any cause will be investigated by the study team and reported to the coordinating center; if appropriate, these will also be reported to the pertinent ethical review boards.

#### CXR reading

All CXR are interpreted by local clinical staff and are classified into one of three categories: normal, abnormal not TB, and abnormal possible TB. CXR are then anonymized and uploaded to the study website. If the CXR is performed using a digital X-ray system, we upload the original digital file. If the CXR is done using an analogic X-ray system (i.e., films), we take photos of the CXR film using standardized procedures and upload these photos.

Once uploaded, the coordinator center in Montreal evaluates each CXR to check if it has the minimum quality criteria. If not, they provide feedback to the sites, and a new CXR is requested. After ensuring the quality criteria, all CXR are forwarded to a panel of experts. They reassess all images for interpretation and categorize them as normal, abnormal not TB, and abnormal possible TB. Feedback to the study team is sent if there is a disagreement between the panel and the clinical staff.

#### Health system costs

All healthcare activities associated with diagnosis of TBI and exclusion of TBD is recorded for each of the three strategies. The healthcare personnel time spent for each of these activities will be estimated from a published time and motion study [24]. Local unit costs will be used to valuate blood tests, images test (such as CXR), and microbiological tests (smear, culture or GX). The costs of healthcare-related visits for all sites will be taken from WHO’s CHOosing Interventions [25]. Healthcare personnel time will be valuated based on average salaries from information provided by facility management in each setting.

#### Patient costs

Patient direct and indirect costs are measured for one household contact per index TB patient. Costs include time and travel costs for visits for investigation and treatment, and all out-of-pocket expenses, particularly for the time, travel, and any other expenses related to TST and CXR (even if the participants do not pay for the actual tests—these may require separate visits to the health facility just to complete them—which requires time and out-of-pocket expenditures).

We have adapted a standardized interviewer-administered questionnaire that we used previously to measure patient and family costs associated with TBD [24]. We have included a small number of items on this questionnaire regarding patient experience and acceptability of study procedures. For participants who completed investigations required for the study but do not require TPT or TBD treatment, the questionnaire is administered within 1 month of completing all investigations. For participants who were recommended to start treatment for TBI or TBD, the questionnaire is applied 3 months after randomization, which should correspond to having been on treatment for about 2 months. We expect that participants will have good recall of their experience with TST injection and reading, performance of CXR, and provision of sputum samples and performance of GX or other microbiologic testing, as 3 months will still be recent enough to avoid recall difficulties. Patient time will be valuated based on an assumption of income equivalent to the average per capita income in the country.

### Sample size

Our primary analysis will address the outcomes in HHC aged 5–50 years of index patients with microbiologically confirmed TB. We will base the total sample size on the proportion of identified HHC who start TPT in each strategy. We are interested in detecting a difference between the proportion of HHC starting TPT in those randomized to the standard algorithm and each of the alternative regimens. The proportion starting TPT during phase 2 of ACT4 [26, 27], at the same study sites, when solutions had been implemented and the barriers of TST and CXR had been resolved, will be used as the likely proportion in the standard arm. We consider that accepting to start treatment will likely be strongly influenced by other household members. We do not have an estimate of the cluster effect of HHC on starting, so we will use the “cluster effect” of study therapy completion which was observed in previous TPT trials in the same sites [28, 29]. This gave an intra-class correlation coefficient (ICC) or clustering effect of HHC of 0.33. Based on this ICC and an average of three HHC aged 5–50 years (observed in the ACT4 trial) [26, 27], we can estimate the design effect as 1 + (household size-1)*ICC. We will assess differences across sites but expect randomization to reduce potential confounders.

In the ACT4 trial [27], 60% of eligible HHC started a TPT regimen once the TST and CXR problems were resolved. We assume that 50% of HHC aged 5–50 would be eligible for TPT and that 60% of eligible HHC contacts in the standard arm would start therapy—for an overall initiation rate of 30% of participants randomized to the standard arm in this trial. To detect an improvement to 85% of eligible starting TPT in the GX arm (strategy 2), resulting in an overall initiation rate of 42.5% in this arm, we would need to enroll 455 participants into each arm. Allowing for 5% withdrawal, or otherwise not analyzable participants, this would inflate the number per arm to 478, so we plan to enroll a total of 1434 participants. If the TPT initiation rate among eligible is 80%, providing an overall initiation rate that is only 10% better than the standard algorithm, then 1371 participants would still provide 60% power to detect a significant difference. Power will be greater if the initiation rate in the standard arm is lower; for example, if only 50% of eligible HHC or 25% overall, initiate TPT, as seen in Table 1. For the 0–5 subgroup, we used the same estimates of effect and ICC but assumed 50% of household with 1 child under 5 and 50% with 2 and a higher loss to follow-up (10%). That would mean a sample size of 284 children under 5. All calculations use alpha = 0.05.

**Table 1:** Sample size required to detect superior initiation of tuberculosis preventive treatment (TPT) with either one of the experimental arms compared to standard arm

TPT initiation proportion among all HHC identified		Number required per group to detect significant difference^a^, accounting for clustering by household		
**Standard**	**Experimental (GX or no TST**)^b^	*N* per arm—80% power	*N* per arm—60% power	Total *N*^c^ (3 arms)
40%	45%	3046	1901	9138
	50%	766	478	2298
35%	40%	2921	1823	8763
	42.5%	1311	818	3933
	45%	742	464	2226
	47.5%	477	298	1431
**30%**	35%	2734	1706	8202
	40%	703	439	2109
	**42.5%**	**455**	**284**	**1365**
	45%	318	199	954
25%	30%	2484	1551	7452
	35%	649	405	1947
	40%	297	186	891

^a^Alpha = 0.05. The intra-class correlation coefficient (ICC) or clustering effect of HHC on TPT initiation was estimated from the ICC for completion in the adult trial comparing 4R with 9H [1], among study subjects who had at least one other family member in the study—i.e., from participants in families of size > 1. We expect the average number of household contacts to be 3 (based on our just completed ACT4 study[2, 3]

^b^In the Standard and GX arms, all children < 5 years and older HHC who are TST positive will be eligible to initiate TPT. We estimate this will be about 50% of all HHC, resulting in the lower overall expected initiation rate among all HHC—as cannot exceed the expected proportion eligible for TPT. In the no TST arm, we expect a higher proportion of HHC will start therapy, but we will estimate the number eligible based on prevalence of positive TST in the same age groups at the same centers in the other two arms; the number required in the no TST arm is therefore the same—based on this estimation

^c^Total is based on 80% power

*Abbreviations*: *CXR* chest X-ray, *TST* tuberculin skin testing, *TB* tuberculosis, *TPT* tuberculosis preventive treatment, *ICC* intra-class correlation coefficient, *HCC* household contacts, *GX* GeneXpert

We expect that significant differences exist between the two involved countries in health systems and patients’ costs. Hence, we have calculated study power for each country (Table 2). For power calculations, costs are based on estimates from the WHO CHOICE database[30] and data gathered as part of prior studies (ACT4) [27]. The number enrolled to each arm (assuming one HHC per household, type 1 error = 0.05) within each country should provide more than 90% power to detect a significant difference in costs.

**Table 2:** Sample size required to detect significant difference in costs between standard and GX arms—in each country

Estimated costs associated with standard^a^ CAD$ 2017			Estimated costs associated with GX^b^ CAD$ 2017			Power to detect effect sizes^d^ (effect size = the detectable difference/SD)		
**Patient’s perspective**^c^	**Health system perspective**	**Total**	**Patient perspective**^c^	**Health system perspective**	**Total**	**0.3**	**0.33**	**0.5**
**Benin**								
20	121	141	16	111	127	0.72	0.8	0.99
**Brazil**								
36	319	355	28	278	306	0.72	0.8	0.99

^a^For standard scenario: We assumed that HHC has two visits for TST (administration and reading). Half have three more visits for medical evaluation and CXR and 20% of these have an added two more visits to collect sputum samples; 25% have all of the above, plus 1 visit for LTBI treatment initiation and 3 more visits for LTBI treatment follow-up

^b^For GX scenario: We assume that HHC has two visits for TST (administration and reading). Half have one more visit for medical evaluation and GX. One quarter also have one visit for LTBI treat initiation and 3 more visits for LTBI treatment follow-up

^c^Costs from the patient perspective: Expenses associated with medical visits assumed to be $4.00 per visit in Benin and $7.50 per visit in Brazil. This accounts for travel costs and additional expenses during travel or at medical visit[4]

^d^To estimate power, we assume alpha = 0.05, and 455/3 = 152 analyzable subjects per group in each country, and we considered the effect size (detectable difference/SD). We do not know the standard deviation but can estimate approximate costs, based on prior work in each country. As an example, based on costs collected previously in Ghana (neighboring country to BENIN, that also participated in our prior RCT of 4RIF vs 9INH) [1], we expect a difference in total costs of $28 between standard and GX arms. If the standard deviation is $84, then the effect size will be ($28/$84) = 0.33. After accounting for clustering by household, assuming an ICC of 0.33 and 4 subjects per household this effect size will result in estimated power of 80%. If the SD is actually smaller (SD = $56), then for the same expected difference in costs, we will have an effect size = 0.5, providing 99% power to detect a significant difference

To enhance recruitment, research staff are present in the participating clinics on all the days that TB patients receive care. In Benin, community health agents also visit index TB patients at home and obtain informed consent from the HHC during home visits. More clinics were added in Brazil after the start of enrolment, to increase recruitment rates.

### Outcome definitions and analysis plan

The primary outcome is the proportion of HHC eligible for TPT who start TPT within 3 months of the index TB patient starting TBD treatment, among all HHC contacts of patients with newly diagnosed index TB patients.

Secondary outcomes include:

- 1. Societal costs (health system and patient costs) of the full cascade-of-care: from initial identification to TPT completion.
- 2. HHC who initiate treatment within 3 months of randomization for microbiologically confirmed or clinically diagnosed TBD that was detected during the contact investigation.
- 3. Prevalence of positive TST (≥ 5 mm or ≥ 10 mm) among all contacts and by age group.
- 4. Incidence of grade 1–4 adverse events related to TPT.
- 5. Completion of TPT—defined as having taken at least 80% of doses in 120% of allowed time.
- 6. Sensitivity and specificity of CXR reading by usual providers in each study site (reference standard will be readings by an external review panel).
- 7. Prevalence of TBD diagnosed using CXR in participants who cannot produce a sputum sample.

#### Primary analysis

We will compare the proportion starting TPT within 3 months of randomization of those eligible for TPT in each experimental arm against the standard arm. The prevalence of positive TST in control and GX arms will be used to estimate the number “eligible” for TBI therapy in all arms. Treatment initiation will be defined as being given a prescription for TPT or dispensed the first month of pills needed for TBI therapy. Since this is a dichotomous outcome, the primary analysis will be a logistic regression, using an identity link, and estimated via generalized estimating equations (GEE) to account for clustering by household. An exchangeable correlation structure and empirical standard errors will be used. The primary outcome analysis will be conducted by a statistician blinded to the allocation group. No interim analysis is planned.

#### Secondary analyses

- 1. Societal costs (health system and patient costs) of the full cascade of care—from initial identification to TBI therapy completion will be compared for each of the two alternate strategies to the standard strategy.
- 2. Prevalence of microbiologically confirmed and clinically diagnosed TBD—detected as part of the initial contact investigation and initiated treatment within 3 months of the date of enrolment will be compared among the three arms.
- 3. Prevalence of positive TST in standard and GX strategies (defined using national guidelines in each country) by age group—0–4, 5–10, 11–17, 18–24, 25–34 years, and older. This is simple descriptive analysis—and will be presented as overall prevalence in the specified age groups, stratified by country.
- 4. Incidence of grade 3–4 adverse events related to TPT. Adverse events are relatively rare outcomes. Poisson regression will be used to compare the occurrence of the adverse events between each of the two experimental arms and the conventional arm. To account for clustering by household, we will use GEE, with an exchangeable correlation structure and empirical standard errors. This secondary outcome analysis will also be conducted by a statistician blinded to the allocation group.
- 5. Completion of TPT—we will use the definition of completion/non-completion of the providers and the TB programs in each country. Since this is a dichotomous outcome, the analysis to compare the proportion completing treatment in each experimental arm against the standard arm will use logistic regression, with an identity link, and estimated via GEE to account for clustering by household, and health facility. An exchangeable correlation structure and empirical standard errors will be used. This secondary outcome analysis will also be conducted by a statistician blinded to the allocation group.
- 6. Sensitivity and specificity of CXR reading by usual providers in each study site. For this analysis, the reference standard will be the readings by the external CXR review
- 7. Potentially missed TBD—defined as treatment initiated for TBD that was detected only as a result of the CXR done in strategy 2 in persons who could not produce a sputum sample for GX.
- 8. All outcomes among HHC of index cases with microbiologically confirmed TB will be compared to those of HHC of index cases with clinically diagnosed TB.
- 9. All outcomes in children < 5 will be compared to outcomes in 5–50 years of age. In this stratum, safety of induced sputum will also be evaluated.

## Trial status

This is protocol version 2 from 1 June 2020. The protocol was registered at clinicaltrials.gov on August 20, 2020 (NCT04528823). The study started on March 27, 2021, in Brazil. In Benin, it started on January 29, 2020, but was interrupted after 2 participants (HHC) were enrolled because of the COVID pandemic; it restarted on March 5, 2021. Recruitment is ongoing in both countries and is expected to terminate on September 30, 2022. Follow-up will last 3 to 9 months after the last recruitment, depending on regimen prescribed. Internal monitoring is carried out 3 times/year by the coordinating center. The median recruitment rate since March 2021 has been 67 (range 33–153) participants monthly.

### Committees

A data safety and monitoring board (DSMB) will be responsible to review any unusual or unexpected events and make recommendations regarding continuing or stopping enrolment to study arms (or the overall study). A trial steering committee will review progress of the ongoing trial, including enrolment and randomization, pragmatic problems such as difficulties with enrolment or withdrawal of consent, as well as need for study amendments.

## Data Availability

The datasets of the current study will be available from the corresponding author on reasonable request 2 years after publication of main results.
